# Urine Endocannabinoids as Novel Non-Invasive Biomarkers for Bladder Cancer at Early Stage

**DOI:** 10.3390/cancers12040870

**Published:** 2020-04-03

**Authors:** Riccardo Vago, Alessandro Ravelli, Arianna Bettiga, Silvana Casati, Giovanni Lavorgna, Fabio Benigni, Andrea Salonia, Francesco Montorsi, Marica Orioli, Pierangela Ciuffreda, Roberta Ottria

**Affiliations:** 1 Urological Research Institute, Division of Experimental Oncology, IRCCS San Raffaele Hospital, 20132 Milano, Italy; vago.riccardo@hsr.it (R.V.); bettiga.arianna@hsr.it (A.B.); lavorgna.giovanni@hsr.it (G.L.); benigni.fabio@hsr.it (F.B.); salonia.andrea@hsr.it (A.S.); montorsi.francesco@hsr.it (F.M.); 2 Università Vita-Salute San Raffaele, 20132 Milano, Italy; 3 Dipartimento di Scienze Biomediche, Chirurgiche ed Odontoiatriche, Sezione di Tossicologia Forense, Università degli Studi di Milano, 20133 Milano, Italy; alessandro.ravelli@unimi.it (A.R.); marica.orioli@unimi.it (M.O.); 4 Dipartimento di Scienze Biomediche e Cliniche L. Sacco, Università degli Studi di Milano, 20157 Milano, Italy; silvana.casati@unimi.it (S.C.); pierangela.ciuffreda@unimi.it (P.C.)

**Keywords:** bladder cancer, endocannabinoid system, endocannabinoid quantification, biomarkers

## Abstract

Due to the involvement of the endocannabinoid system (ECS) in cancer onset and progression and the less studied connection between ECS and bladder cancer, here an evaluation of the ECS modifications associated with bladder cancer is reported. Urine samples were collected from healthy volunteers and patients with bladder cancer at different grades. Endocannabinoids (ECs) and N-acylethanolamides (NAEs) were quantified by HPLC-MS/MS and results normalized for creatinine content. An increase in the urine concentrations of four ECs and NAEs analyzed was observed with a statistically significant increase in the arachidonoylethanolamide (AEA) and stearoylethanoamide (SEA) associated with bladder cancer. Receiver operating characteristic curves built with AEA and SEA data allowed the selection of 160 pg/mL for SEA (area under the curve (AUC) = 0.91, Selectivity (SE) 94%, Specificity (SP) 45%) and 8 pg/mL for AEA (AUC = 0.85, SE 94%, SP 61%) as the best cut-off values. Moreover, data from bladder cancer samples at different grades were derived from The Cancer Genome Atlas, and the expressions of thirteen different components of the “endocannabinoidome” were analyzed. Statistical analysis highlights significant variations in the expression of three enzymes involved in EC and NAE turnover in bladder cancer.

## 1. Introduction

**Figure 1:**
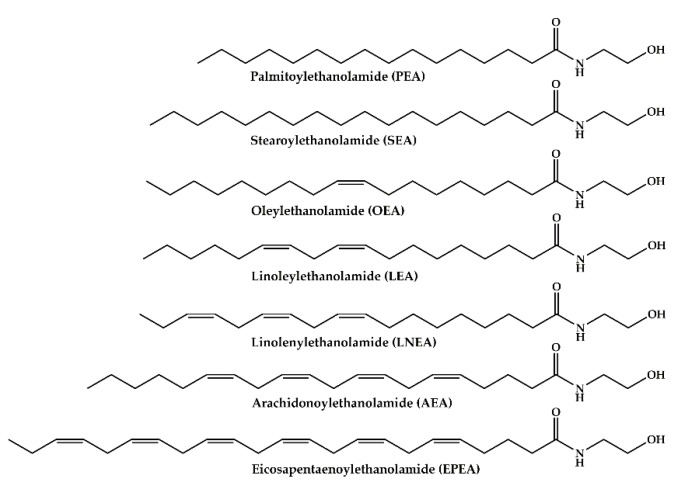
Chemical structures of endocannabinoids (ECs) and N-acylethanolamides (NAEs) analyzed in urine samples.

Bladder cancer is the ninth most frequently diagnosed cancer worldwide, with incidence rates that are consistently lower in women than men. The highest rates are observed in men in Southern and Western Europe and North America [1] with 80,470 new cases in 2019; the estimated number of deaths in the USA was 17,670 [2]. In the European Union, the estimated number of new bladder cancer cases in 2018 was 19,710, with an estimation of 65% deaths [3]. Bladder cancer has the highest lifetime treatment costs per patient of all cancers, and costs have increased steadily since 1996. Despite significant improvements in preventing disease progression and improving survival, bladder cancer is characterized by a high rate of tumor recurrence and potential progression, regardless of treatment with surgery, chemotherapy, or immunotherapy [4]. Patients with bladder cancer can be divided into patients with muscle-invasive bladder cancer (MIBC, Stages II to IV), disease that mainly requires radical surgical treatment and chemoradiation, and patients with the less severe non-muscle-invasive bladder cancer (NMIBC, Stage I). For NMIBC, long-term surveillance with periodic cystoscopy is often required with heavy effects on patients’ quality of life [5]. Moreover, the high recurrence rate and ongoing invasive monitoring requirements are the key contributors to the economic and human toll of this disease. It has become clear that new, less invasive diagnostic and prognostic methods for bladder cancer surveillance are needed. The great advantage of monitoring urological tumors is the option to exploit urine samples, which are easy to retrieve and are actually enriched in cancer-cell-derived molecules due to direct contact with the tumor itself. The possibility of discovering novel diagnostic markers for bladder cancer in urine could overcome the continuous, invasive monitoring of patients. The endocannabinoid system (ECS) is an endogenous signaling system that is transiently active. The biological effects of endocannabinoids (ECs), endogenous long-chain fatty acid derivatives able to activate the ECS, depend on their trafficking in extracellular space and lifespan, which is limited by enzymatic degradation. The ECS includes ECs, their specific receptors (cannabinoid receptors 1 and 2 (CB1 and CB2)) and biosynthetic (N-acyl-phosphatidylethanolamine lipase-D and diacyl glycerol lipase) and catabolic pathways (fatty acid amide hydrolase (FAAH), monoacylglycerol lipase (MGLL), and N-acylethanolamine acid amide hydrolase (NAAA)) [6,7]. Arachidonoylethanolamide (AEA) is the earliest EC described and belongs to the group of N-acylethanolamides (NAEs), which comprises other long-chain fatty acid ethanolamides and non-endocannabinoids, such as palmitoylethanolamide (PEA) and oleoylethanolamide (OEA). PEA and OEA, although lacking affinity for CB1 and CB2, can potentiate the effects of AEA via an “entourage effect” at its targets or compete with AEA for the enzymes involved in its metabolism. Moreover, PEA and OEA can also activate transient receptor potential vanilloid 1 (TRPV1) channels as well as the nuclear peroxisome proliferator-activated receptors (PPARs). Together with the ECS, some NAEs, such as OEA and PEA, and their metabolic enzymes and targets, constitute a large part of the “endocannabinoidome”. ECs and NAEs are a family of lipid mediators involved in a wide range of biological effects [8]. It has been demonstrated that the ECS is functionally expressed in the human bladder [9] and that its regulation is implicated in lower urinary tract function and dysfunction [10]. Moreover, recent literature strongly suggests a role for the ECS in the pathogenesis of cancer [11,12], describing the involvement of ECs and NAEs in maintaining balance in cell proliferation. It has been shown successfully that ECs can act by several different cellular mechanisms, including inhibition of cell proliferation, migration and progression; inhibition of angiogenesis; and promotion of apoptosis and/or cell cycle arrest [13,14,15,16]. In addition, the EC involvement in bladder cancer proliferation and progression has been demonstrated in ECV304 [17], RT4, RT112 [18], 5637, and HT-1376 [19] cellular models resembling different tumor grades. Starting from this knowledge, a possible modification in ECs and NAEs levels in urine from patients with bladder cancer can be hypothesized. Here we report for the first time the HPLC-MS/MS quantification of seven ECs and NAEs in urine from healthy subjects and patients affected by bladder cancer at different stages. In particular, the levels of AEA, PEA, OEA, linoleylethanolamide (LEA), linolenoylethanolamide (LNEA), stearoylethanolamide (SEA), and eicosapentaenoylethanolamide (EPEA) (Figure 1) were analyzed in urine of healthy subjects and bladder cancer patients with the aim of revealing novel and possible non-invasive biomarkers for bladder cancer. Moreover, since 2005, The Cancer Genome Atlas (TCGA) Pilot Project has generated a multi-platform molecular profile of more than 11,000 human tumors across 33 different cancer types [20] and has collected survival data from 11,600 patients [21], allowing the association between key genomic changes and clinical prognosis to be analyzed. Aiming to reveal significant changes in the ECS associated with bladder cancer outcome and progression, the expression and prognostic impact of 13 components of the “endocannabinoidome” were explored in 404 bladder cancer samples, using high-dimensional datasets of cancer specimens from clinical patients in The Cancer Genome Atlas.

## 2. Results

### 2.1. EC and NAE Quantification in Urine

PEA, SEA, OEA, AEA, LEA, LNEA, and EPEA (Figure 1), seven different ECs and NAEs, were quantified by HPLC-MS/MS analysis in urine samples from healthy volunteers and patients with bladder cancer, whose characteristics are displayed in Table 1. Caucasian patients with a diagnosis of primary urothelial carcinoma of the bladder were enrolled in the study. Patients with concomitant or previous diagnoses of prostate, renal, and upper excretory tract cancer, urinary tract infections, and kidney failure were excluded. Control urine samples were obtained from healthy volunteers without any current or previous diagnosis of cancer or other pathologies of the urinary tract.

**Figure 2:**
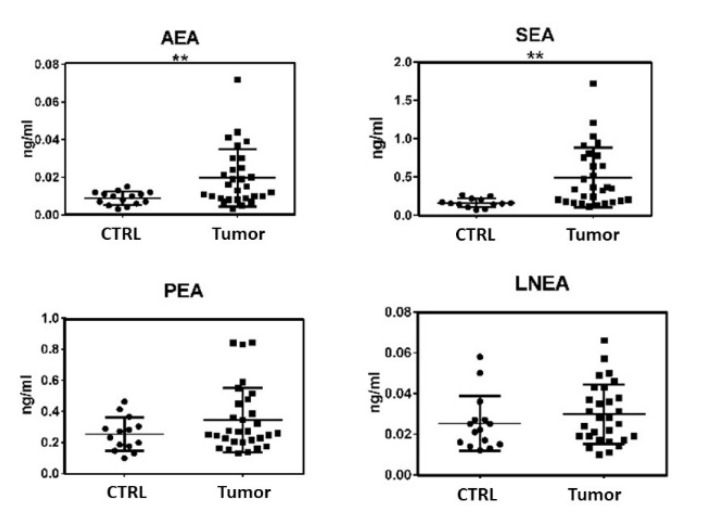
Levels of the ECs and NAEs in urine samples, expressed in ng/mL: arachidonoylethanolamide (AEA), N-palmitoylethanolamide (PEA), N-stearoylethanolamide (SEA), and N-linolenoylethanolamide (LNEA). ECs and NAEs were quantified by HPLC-MS/MS analysis in urine samples of healthy volunteers (CTRL) and patients with bladder cancer (Tumor). The statistical significance of differences was evaluated by the Student’s t-Test, ** *p* ≤ 0.01.

The obtained data were then normalized with creatinine content to exclude the influence of the urine dilution factor. As shown in Figure 2, only AEA, SEA, PEA, and LNEA quantifications gave reliable results. EPEA and OEA urine contents, indeed, were too low for detection and quantification, respectively.

**Figure 3:**
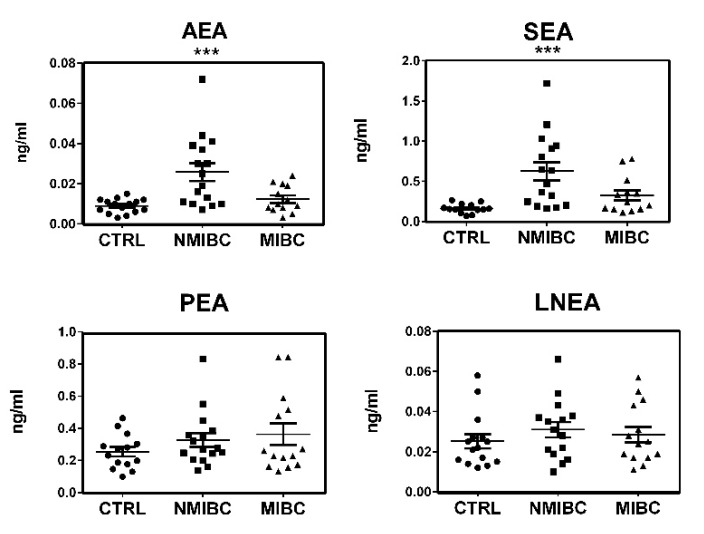
Levels of the ECs and NAEs in urine samples, expressed in ng/mL, dividing patients between non-muscle-invasive and muscle-invasive bladder cancer: arachidonoylethanolamide (AEA), N-palmitoylethanolamide (PEA), N-stearoylethanolamide (SEA), and N-linolenoylethanolamide (LNEA). ECs and NAEs were quantified by HPLC-MS/MS analysis in healthy volunteer (CTRL) and bladder cancer patients with non-muscle-invasive bladder cancer (NMIBC) and muscle-invasive bladder cancer (MIBC). The statistical significance of differences was evaluated by one-way ANOVA, with *** *p* ≤ 0.001.

LEA levels, instead, showed too much subject–subject variability to be considered. From the four remaining ECs and NAEs, despite the increment in the four ECs and NAEs concentrations reported in Figure 2, only AEA and SEA displayed a statistically significant increase in tumor patients’ samples (Figure 2). When data were split between NMIBC and MIBC, although PEA and LNEA levels did not show significant variations, again a constant increase in the concentrations of these molecules in patients’ urine was evident (Figure 3). For compounds AEA and SEA, one-way ANOVA analysis showed that statistically significant differences were maintained.

**Figure 4:**
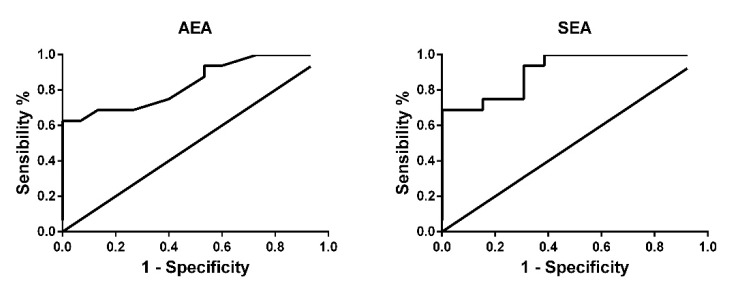
Univariate ROC curve analysis of AEA and SEA. Urine marker levels were assessed for their ability to discriminate bladder cancer patients from healthy subjects.

The diagnostic performance for the proposed biomarkers was evaluated using receiver operating characteristic (ROC) analysis, and the obtained ROC curves, built with AEA and SEA data, are shown in Figure 4.

Moreover, the data obtained from ROC analysis with AEA and SEA values are reported in Table 2. The calculated area under the curve (AUC) values were greater or equal to 0.85 for both AEA and SEA. The cut-off values proposed were selected to obtain the best possible values for both sensitivity and specificity (Table 2) and are 8 pg/mL for AEA and 160 pg/mL for SEA.

### 2.2. The Cancer Genome Atlas Analysis

To better elucidate the role of the ECS in bladder cancer and the results obtained from urine, we took advantage of the cohort of patients with bladder cancer available on The Cancer Genome Atlas (TGCA) database. We analyzed the gene expression data of the “endocannabinoidome”, which include enzymes involved in ECs synthesis (1-phosphatidylinositol 4,5-bisphosphate phosphodiesterase beta-1-4 (PLCB1-4), protein tyrosine phosphatase N22 (PTPN22), αβ hydrolase D4 (ABHD4), glycerophosphodiesterase-1 (GDE1), sn1-specific diacylglycerol lipase alpha (DAGLA), sn1-specific diacylglycerol lipase beta (DAGLB), and N-arachidonoyl-phosphatidyl-ethanolamine phospholipase D (NAPE-PLD)) as well as those in ECs catabolism (FAAH, NAAA, and MGLL). Patients with bladder cancer were divided into four groups based on the tumor stage (Table 3). The comparison of the four groups showed statistically significant differences in DAGLA, NAAA, and, particularly, FAAH.

**Figure 5:**
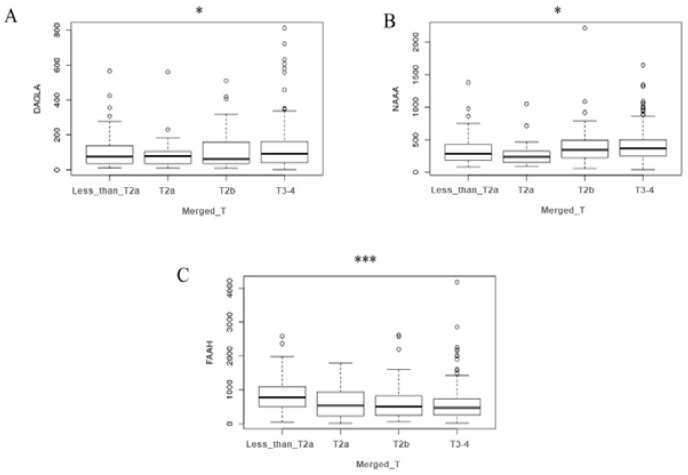
Analysis of endocannabinoid system components expression in patients with bladder cancer. In silico gene expression analysis of DAGLA (**A**), NAAA (**B**), and FAAH (**C**) throughout bladder cancer stages from TGCA dataset. Data are expressed as reads per kilobase million (RPKM) and box-and-whisker plots showing minimum, 25th percentile, 50th percentile (median), 75th percentile, and maximum values of the percentage. The expression difference among stages was evaluated by performing an ANOVA test. * *p* < 0.5; *** *p* < 0.001.

While NAAA and FAAH showed peculiar behavior going from lower to higher stages, with the first exhibiting an increasing pattern and the second a decreasing one, a clear tendency of DAGLA was not definite (Figure 5).

**Figure 6:**
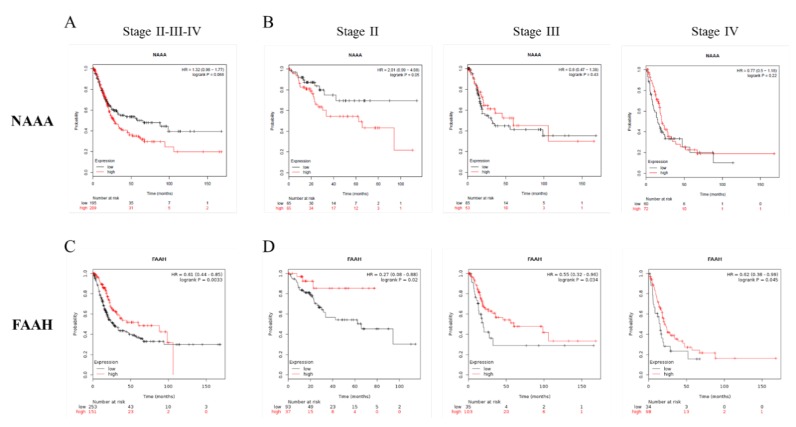
High NAAA and low FAAH levels correlate with poor prognosis. Kaplan–Meier plots of patients with bladder cancer were drawn considering the high or low NAAA and FAAH gene expression levels on the overall population (**A** and **C**) or on the single stage (**B** and **D**).

With the aim of better understanding the impact of the FAAH and NAAA expressions on bladder cancer development, we split patients into two groups—high and low expressors of these enzymes. The high expression of NAAA is correlated to a drastically reduced overall survival in patients with bladder cancer, in particular in Stage II (Figure 6A,B). On the other hand, low levels of FAAH are linked to poor prognosis (Figure 6C,D), especially at Stages II and III.

## 3. Discussion

The alteration in the ECS activity during tumor onset and progression and its involvement in bladder cancer has been demonstrated in cellular models [17,18,19]. On the other hand, to date no data are available on the quantification of ECs and NAEs levels in the urine of healthy subjects or patients with bladder cancer. Bladder cancer has a high rate of recurrence leading to long-term surveillance with periodic invasive cystoscopy. In this preliminary study, we explored the option of exploiting urine samples, which are naturally enriched in cancer-cell-derived molecules, in order to discover novel diagnostic markers for bladder cancer that could lead to long-term non-invasive surveillance of patients with bladder cancer. With this aim, PEA, SEA, OEA, AEA, LEA, LNEA, and EPEA were quantified in urine samples of healthy volunteers and patients with bladder cancer by HPLC-MS/MS analysis. To exclude the possibility that differences in ECs and NAEs levels could be due to urine dilution, the creatinine content of urine samples was measured by Jaffe’s reaction [22] and the obtained results were used for data normalization. From ECs and NAEs analyses, EPEA was undetectable in all samples, both from healthy subjects and patients with bladder cancer, while OEA levels were under the limit of quantification of the applied analytical method in all samples. Moreover, LEA quantification unfortunately highlighted too much high variability in the two populations assessed to be considered for further statistical analysis and consideration. For these reasons, these three molecules have been excluded from the discussion. The remaining four molecules represent the highest amount of ECs and NAEs, of the analyzed lipid derivatives, found in urine samples, characterized by the following distribution in healthy subjects: PEA—48%, SEA—31%, LNEA—5%, and AEA—17%. These results reveal a predominance of saturated fatty acid derivatives. The comparison with data from patients with bladder cancer highlights a modification in the ECs and NAEs profile linked to pathology along with a relative discrepancy in lipid composition. In particular, we observed a percentage decrease for PEA (33%) and LNEA (3%) and a percentage increase for SEA (47%) and AEA (18%). Although there were some noticeable changes in the ECs and NAEs profile, with an increase in the concentration of all four (PEA, LNEA, SEA, and AEA), only the amounts of AEA and SEA were increased in a statistically significant way in patients with bladder cancer, as shown in Figure 2.

The deregulation of AEA in cancer disease is not surprising considering that anandamide was the first ECs to be described in literature and the best studied in pathological conditions, in particular in cancer disease [11,12]. Considering its anti-carcinogenic activity, demonstrated in gliobastoma [23], uterine cervix [24], and neuroblastoma, lymphoma, and breast cancer [25], the enhanced levels of AEA in the urine of patients with bladder cancer can be due to a mobilization of the ECS to counteract cancer cell proliferation. Of particular interest is the increase of SEA, the ethanolamide of the saturated stearic acid, a very little studied NAEs. Although SEA cannot be considered an ECs because its activity is not mediated by CB receptors, it can be considered an entourage compound of the ECS. Indeed, it has been demonstrated by Maccarone and co-workers [26] that SEA is hydrolyzed by FAAH and is able to potentiate the activity of AEA. Moreover, the SEA cannabimimetic and pro-apoptotic properties have been demonstrated in C6 glioma cells [27] and tumoral human brain cells [28]. When the data were split between non-muscle-invasive (Stage I) and muscle-invasive (Stages II and III) bladder cancers, all four ECs and NAEs displayed a similar trend with an increase in ECs and NAEs concentrations and the statistical significance was maintained for AEA and PEA, as highlighted by one-way ANOVA analysis (Figure 3). In this case, the small number of patients (14 and 16 for the two groups, respectively) and the variability intra-group did not allow statistical significance in the comparison of each group with the control group to be obtained. The NMIBC group seemed to show an increase in AEA and SEA in respect to MIBC but also variance in this group was augmented, which is in part due to a single case that displayed quite high values (but not enough to be considered an outlier); for this reason these results do not allow us to draw unequivocal conclusions. Together these results seem to point out an activation of the ECS in response to bladder cancer onset, prompting us to investigate the possibility of promoting AEA and SEA as urine biomarkers for bladder cancer. Even if the number of bladder cancer patients (30) can be considered too small for ROC analysis, we decided to perform it anyway and to report the obtained results in order to have a preliminary evaluation of the possibility of proposing these two ECs as future biomarkers. Both AEA and SEA display the ability to discriminate bladder cancer patients from healthy subjects, with an AUC value of 0.85 for AEA and 0.91 for SEA. Selecting 8 pg/mL for AEA and 160 pg/mL for SEA as cut-off values, the sensitivities for diagnosing bladder cancer based on the AEA and SEA levels in urine are 94% for both, and the specificities are 45% and 61%, respectively. These two cut-off values were selected to obtain good sensitivity, and we can suppose that the low specificity could be overcome by considerably increasing the number of patients. These results encourage us to propose AEA and SEA levels in urine as potential biomarkers preventively examined for individuals at high risk of bladder cancer recurrence to determine the necessity of cystoscopy examination. Additional experiments involving a great number of patients are required to confirm our hypothesis. Moreover, a large-scale study could also reveal statistically significant variations in the other ECs and NAEs levels. To better understand the role of ECs and NAEs in bladder cancer, we analyzed gene expression data from TGCA database regarding the metabolic pathway of the ECS, comprising the synthetic enzymes (PLCB1-4, PTPN22, ABHD4, GDE1, DAGLA, DAGLB, and NAPE-PLD) and the hydrolytic ones (FAAH, NAAA, and MGLL). The division of TGCA data into four groups, based on the tumor stage, allowed us to highlight the statistically significant variation in the expression of only one synthetic enzyme, the sn1-specific DAGLB, which increases with the progression of the tumor stage (from T2b to T3–T4, Figure 5). Considering the hydrolytic enzymes, a particular behavior is shown by NAAA and FAAH with an apparent decrease in the expression of the first at stage T2a followed by an increase from the T2b grade, while the second shows an evident decrease from stage T2a to T3-4 of bladder cancer as reported in Figure 5. Moreover, splitting patients into two groups with high or low expression of these two enzymes, the high expression of NAAA correlates to drastically reduced survival, in particular at Stage II, while low levels of FAAH correlate to poor prognosis. Taken together, these results highlight a modulation of the activity of the ECS in bladder cancer, supporting the hypothesis of an activation of the ECS toward bladder cancer. Further studies aimed at analyzing in depth the mechanistic aspect of the ECS activation in bladder cancer will help us to understand the involvement of this lipid signaling system in bladder cancer.

## 4. Materials and Methods

### 4.1. Reagents and Solvents

PEA, C16:0; SEA, C18:0; OEA, C18:1; AEA, C20:4; LEA, C18:2; LNEA, C18:3; EPEA, C20:5; *d*_4_-SEA; and *d*_4_-LNEA were synthesized and completely characterized in our laboratories as previously described [29,30,31,32]. HPLC grade and LC-MS grade organic solvents were purchased from Panreac Quimica Sau (Barcelona, Spain). Acetonitrile (HPLC grade) and formic acid (used for elution of the analytes), NaOH, and picric acid were purchased from Sigma-Aldrich (Munich, Germany). Solid-phase extraction cartridges OASIS HBL (30 mg/mL) were purchased from Waters (Etten-Leur, The Netherlands).

### 4.2. Patients Selection and Urine Collection

Thirty patients aged 40–81 years (Table 2) were enrolled in the present study. All the studies carried out on patients’ samples were approved by the Institutional Ethical Committee (Ospedale San Raffaele, Milan, protocol URBBAN, Rev. 2 February 2014, approval date 3 March 2014), and the specific informed consent was obtained. All the experimental procedures involving human biological material were carried out in compliance with the approved guidelines. Control urine samples were obtained from healthy volunteers aged 45–59 years without any current or previous diagnoses of cancer or other pathologies in relation to the urinary tract.

Caucasian patients aged between 40 and 81 years with a diagnosis of primary urothelial carcinoma of the bladder were recruited. Patients with concomitant or previous diagnoses of prostate, renal and upper excretory tract cancer, urinary tract infections, and kidney failure were excluded. Urine samples were collected before the surgical intervention and processed soon after. The samples were centrifuged at 300g for 5 min, aliquoted, and stored at −80 °C until use.

### 4.3. EC and NAE Quantification in Urine

ECs and NAEs were quantified on a ABSciex 5600 TripleTOF mass spectrometer (AB Sciex, FosterCity, CA, USA) coupled with an Agilent 1200 Infinity pump LC system equipped with an Agilent 1290 Infinity autosampler (Agilent Technologies, Waldbronn, Germany) using the HPLC-MS/MS method previously reported [32,33,34]. Briefly, 1.0 mL of urine was spiked with internal standard (*d*_4_-SEA and *d*_4_-LNEA) and 800 µL of cold acetone was added for protein precipitation. After centrifugation, the supernatants were purified by a solid-phase extraction (SPE) procedure with a manifold SPE extractor and OASIS HBL (30 mg/mL). After conditioning of the cartridge (1.0 mL methanol/water, 60:40), the sample was loaded and the cartridge washed (1.0 mL methanol/water, 60:40). Then the cartridge was left for 1 h to dry under vacuum, and the elution was performed with 750 µL of acetonitrile, containing 1% of formic acid. Eluates were dried under vacuum, reconstituted in 50 µL of acetonitrile, containing 1% of formic acid, and analyzed. Quantifications were performed using Multiquant 1.2.1 software by AB Sciex. All extraction and purification experiments were performed in duplicate, and all eluates were injected (20 µL) and analyzed twice; the means of the obtained values have been used for the statistical analyses.

### 4.4. Creatinine Quantification in Urine

The creatinine concentration in the urine samples was determined by Jaffe’s reaction as described in literature [22]. Briefly, the urine samples were diluted 100 times in distilled water, then 1% NaOH and 1% picric acid were added and the obtained solutions were mixed and incubated at room temperature for 15 min; after that, the absorbance was read at 520 nm. In a typical analysis in a 96-well plate, in each well, 100 µL of diluted urine, 25 µL of NaOH 0.25N, and 25 µL of picric acid (1% in water) were added. Each urine was analyzed in triplicate and the mean value was used for normalization.

### 4.5. The Cancer Genome Atlas Analysis

For gene expression analysis, normalized RPKM (reads per kilobase million) values were downloaded from the Genomics Data Common Data Portal (https://portal.gdc.cancer.gov/) along with patient clinical data. TGCA dataset includes 404 bladder cancer patients (74% male and 26% female) with different stages of the disease as follows: less than T2a (42 patients), T2a (25 patients), T2b (56 patients), and T3–T4 (251 patients). The vast majority of the tumors are high grade (94%), while low grade tumors are 5%. Patients without indications about the tumor stage or grade were excluded. ANOVA tests were performed by using the R software package (https://www.R-project.org/) [35,36]. Kaplan–Meier survival plots of bladder cancer patients with high or low FAAH and NAAA were drawn using data from the Kaplan–Meier database available at www.kmplot.com [37].

### 4.6. Statistical Analysis

All urine samples (from controls and patients) were extracted in duplicate and analyzed (at least) in duplicate; the mean calculated for each sample was used for statistical analyses. Statistical analyses were performed with GraphPad Prism 5; the significance of the EC and NAE level variations between the samples of the controls and the patients were assessed by the unpaired Student’s t-test. A one-way ANOVA was applied to compare healthy controls to patients with bladder cancer divided into two groups—NMIBC and MIBC. The diagnostic performance for each biomarker was evaluated using receiver operating characteristic curve (ROC) analysis, reporting the area under the curve (AUC). The significance of differences in the endocannabinoid system components expression in healthy and bladder cancer samples were assessed by one-way ANOVA using R software.

## 5. Conclusions

Bladder cancer is a frequently diagnosed cancer worldwide with a high rate of recurrence linked to significant lifetime treatment costs and long-term surveillance with invasive monitoring. Moreover, the involvement of the ECS in urinary cancer onset and progression is well known. Considering the urgent need of a new and non-invasive monitoring process and the advantageous option of being able to exploit urine samples, which are directly in contact with tumor cells, we explored the possibility of linking ECS activity alterations to bladder cancer onset.

The obtained preliminary data strongly suggest promoting ECs and NAEs, with particular attention to AEA and SEA urine levels, as early biomarkers for bladder cancer. Moreover, statistically significant variations in the expression of EC metabolic enzymes is linked to bladder cancer, supporting the hypothesis of an activation of the ECS to counteract bladder cancer.

Further experiments, performed on a larger cohort of patients, are required to validate this hypothesis and could promote ECs and NAEs as new biomarkers.

## Figures and Tables

**Table 1:** Demographic and clinical–pathological characteristics of studied patients with bladder cancer and healthy controls.

Variables	NMIBC pTa-pT1 (*n* = 16)	MIBC Pt2-Pt4 (*n* = 14)	Healthy Control (*n* = 14)
**Age** (mean ± SD, years)	65.0 ± 17.2	65.0 ± 11.4	57.2 ± 11.9
**Tumor grade**			
Low grade (G1–G2)	13 (81%)	0 (0%)	
High grade (G2–G3)	3 (19%)	14 (100%)	
**Carcinoma in situ (CIS)**			
Yes	2 (12%)	9 (64%)	
No	14 (88%)	5 (36%)	
**Lymph node involvement**			
N0	16 (100%)	8 (57%)	
N1–3	(0%)	6 (43%)	
**First episode**			
Yes	7 (44%)	3 (21%)	
No	9 (56%)	11 (79%)	
**Angioinvasion**			
Yes	0 (100%)	4 (28%)	
No	16 (0%)	10 (72%)	

**Table 2:** Bladder cancer diagnosis values for AEA and SEA.

Markers	Sensitivity	Specificity	Cut-off	Likelihood	AUC
AEA	94%	45%	8 pg/mL	1.76	0.85
SEA	94%	61%	160 pg/mL	2.43	0.91

**Table 3:** Gene expression of the endocannabinoid system components in bladder cancer samples from The Cancer Genome Atlas (TGCA) dataset. 1-phosphatidylinositol 4,5-bisphosphate phosphodiesterase beta-1-4 (PLCB1-4), N-arachidonoyl-phosphatidyl-ethanolamine phospholipase D (NAPE-PLD), protein tyrosine phosphatase N22 (PTPN22), αβ hydrolase D4 (ABHD4), glycerophosphodiesterase-1 (GDE1), sn1-specific diacylglycerol lipase alpha (DAGLA), sn1-specific diacylglycerol lipase beta (DAGLB), fatty acid amide hydrolase 1 (FAAH), N-acylethanolamine acid amidase (NAAA), and monoacylglycerol lipase (MGLL) values were reported by stage. The statistical significance of differences, highlighted in bold, was evaluated by ANOVA. * *p* ≤ 0.05, *** *p* ≤ 0.001.

Gene Name	less_than_T2a	T2a	T2b	T3–T4	*p* Value
**PLCB1**	343.60	430.09	334.07	364.06	0.251
**PLBC2**	232.18	430.09	258.14	271.75	0.702
**PLBC3**	1986.26	2095.16	2065.71	2045.11	0.900
**PLBC4**	89.60	130.41	131.73	122.30	0.720
**NAPE-PLD**	398.99	382.45	324.87	337.41	0.085
**PTPN22**	42.53	43.75	71.75	67.90	0.179
**ABHD4**	1030.56	924.14	923.84	1055.02	0.154
**GDE1**	3131.74	2719.12	2513.97	2694.98	0.475
**DAGLA**	109.29	94.03	114.13	124.48	0.023 *
**DAGLB**	587.9	530.75	652.54	624.55	0.138
**FAAH**	886.46	612.25	650.59	577.65	1.00 × 10^−4^ ***
**NAAA**	334.28	269.56	415.36	405.78	0.012 *
**MGLL**	684.47	807.24	842.41	957.37	0.158
